# Validation of wearable visual feedback for retraining foot progression angle using inertial sensors and an augmented reality headset

**DOI:** 10.1186/s12984-018-0419-2

**Published:** 2018-08-15

**Authors:** Angelos Karatsidis, Rosie E. Richards, Jason M. Konrath, Josien C. van den Noort, H. Martin Schepers, Giovanni Bellusci, Jaap Harlaar, Peter H. Veltink

**Affiliations:** 1 Xsens Technologies B.V, Pantheon 6, Enschede, 7521 PR The Netherlands; 2 Department of Biomedical Signals and Systems (BSS), Technical Medical Centre, University of Twente, Enschede, The Netherlands; 3 Department of Rehabilitation Medicine, Amsterdam Movement Sciences, VU University Medical Center, Amsterdam, The Netherlands; 4 Academic Medical Center, Musculoskeletal Imaging Quantification Center (MIQC), Department of Radiology and Nuclear Medicine, Amsterdam Movement Sciences, Amsterdam, The Netherlands; 5 Department of Biomechanical Engineering, Delft University of Technology, Delft, The Netherlands

**Keywords:** Foot progression angle, Inertial sensors, Real-time biofeedback, Augmented reality headset, Gait retraining, Knee osteoarthritis

## Abstract

**Background:**

Gait retraining interventions using real-time biofeedback have been proposed to alter the loading across the knee joint in patients with knee osteoarthritis. Despite the demonstrated benefits of these conservative treatments, their clinical adoption is currently obstructed by the high complexity, spatial demands, and cost of optical motion capture systems. In this study we propose and evaluate a wearable visual feedback system for gait retraining of the foot progression angle (FPA).

**Methods:**

The primary components of the system are inertial measurement units, which track the human movement without spatial limitations, and an augmented reality headset used to project the visual feedback in the visual field. The adapted gait protocol contained five different target angles ranging from 15 degrees toe-out to 5 degrees toe-in. Eleven healthy participants walked on an instrumented treadmill, and the protocol was performed using both an established laboratory visual feedback driven by optical motion capture, and the proposed wearable system.

**Results and conclusions:**

The wearable system tracked FPA with an accuracy of 2.4 degrees RMS and ICC=0.94 across all target angles and subjects, when compared to an optical motion capture reference. In addition, the effectiveness of the biofeedback, reflected by the number of steps with FPA value ±2 degrees from the target, was found to be around 50% in both wearable and laboratory approaches. These findings demonstrate that retraining of the FPA using wearable inertial sensing and visual feedback is feasible with effectiveness matching closely an established laboratory method. The proposed wearable setup may reduce the complexity of gait retraining applications and facilitate their transfer to routine clinical practice.

## Background

Knee osteoarthritis (KOA) is a leading cause of disability in the elderly population [1]. To date, there is no cure available for the disease and treatment options are of pharmacological, surgical or biomechanical nature [2–4]. Pharmacological treatments alleviate only the symptoms (pain, discomfort and swelling), while surgical treatments usually involve total knee replacement and are only considered in severe stages of the disease [5]. Biomechanical interventions are conservative non-pharmacological treatments, which aim at decreasing or distributing the loading across the knee joint. This mechanical joint loading has been related to cartilage degeneration, pain, and disease progression [6].

A common biomechanical treatment is gait retraining. Through these treatments patients learn and gradually adopt a modified gait pattern that results in decreased loading across the knee joint. [7, 8]. The training is typically achieved by tracking the body biomechanics and using this information to drive a real-time feedback modality, such as a vibration, an audio sound or a visualization pattern [9]. An advantage of gait retraining compared to other biomechanical treatments, such as use of wedge insoles, knee braces or canes, is that it does not require any additional devices to alter gait mechanics.

Knee joint loading can be quantified through the medial and lateral tibiofemoral contact forces [10]. Due to practical difficulties in measuring the internal knee contact forces in a non-invasive manner, the net knee joint moment has been considered a convenient surrogate measure [11]. However, instructing patients to decrease a complex kinetic parameter, such as the knee joint moment, in real-time, has been shown to be less effective than explicitly instructing the correct movement that achieves the decrease in the loading [12]. These findings are in line with previous studies which showed that higher reduction in the knee adduction moment can be achieved by altering related kinematic parameters, such as the foot progression angle (FPA) [13–20].

Despite the demonstrated benefits of gait retraining, it is currently not used in clinical practice [21]. One of the primary reasons impeding clinical adoption is the expensive, complex, time-consuming, and space-bound instrumentation that is required to accurately assess the biomechanical parameters. Conventionally, a gait laboratory is utilized, in which multiple cameras track the three-dimensional positions of skin-mounted passive or active markers. Next, the segment positions and orientations are assessed through computational techniques such as direct or inverse kinematics [22, 23].

An ambulatory alternative to the lab-bounded measurement systems is composed of inertial measurement units (IMUs) that can derive orientation of a sensor in space [24]. Specifically, fusion of the accelerometer, gyroscope, and magnetometer signals and incorporation of a biomechanical model and external contact updates enable consistent drift-free motion capture [25, 26]. In addition, IMU-based systems are typically low cost, low power, highly portable, minimally obstructive, easily wearable, acceptable by older adults and therefore comprise an ideal alternative to facilitate the clinical translation of movement analysis systems. Despite their potential, to date, the use of IMUs in gait retraining applications for KOA has only received limited attention [27, 28]. Exploiting the advantages of IMUs, featuring high performance and applicability, may remove the complexity of the current laboratory approaches, decrease the costs, and make treatments available to a larger number of patients.

Besides motion tracking, the second component required in gait retraining is the biofeedback. In a recent systematic review, studies using laboratory-based biofeedback to target knee joint loading either directly or indirectly were analyzed [29]. Most studies used visual feedback modes [7, 12, 20, 30–36] or multi-modal visual-tactile [37, 38], and less often solely tactile [19, 39–41] or auditory feedback [42]. Another review focusing on wearable sensing and feedback techniques reported that until recently, most studies utilizing wearable feedback incorporated primarily tactile modalities [9]. These devices are typically unobtrusive, but they act as on/off switches that can only convey binary information to the user. In addition, tactile feedback was reported to require longer training times for patients to converge to a target pattern, compared to visual feedback. Wheeler et al. [37] Wearable visual feedback was until recently challenging due to practical limitations. The conceptual and technical feasibility of wearable visual feedback for knee joint angle using two IMUs and a small screen on a smart-glass was demonstrated by Steuner et al. [43]. Recent advances in augmented reality (AR) headsets, such as the Microsoft HoloLens [44], allow the projection of virtual objects on the user’s field of view, via head-worn screens. As a result, the wearable biofeedback setups can be enriched with quantitative information, which can not only convey whether the user is achieving the desired target range, but also quantify the difference from the target.

The overarching objective of this study was to develop and evaluate a wearable biofeedback system for gait retraining purposes, as an alternative to currently existing lab-bound setups. In order to achieve this, the first objective was to develop a wearable real-time visual feedback driven by FPAs calculated using input from a commercially available inertial motion capture system; utilizing accelerometers, gyroscopes, and magnetometers. We hypothesized that the proposed wearable system would provide accurate assessments in timing and magnitudes of the FPA when compared to a conventional optical motion capture laboratory setup. The second objective was to evaluate the feedback effectiveness of the wearable system reflected by the number of steps with FPA within a defined target range. We hypothesized that participants would perform equally well in achieving the desired FPAs using the wearable system, when compared to the established laboratory setup. It is envisioned that the proposed wearable setup may reduce the complexity of gait retraining and facilitate their transfer into routine clinical practice.

## Methods

### Subjects

Eleven (11) healthy volunteers (4 males, 7 females, age: 28.26±4.55 years; height: 1.78 ±0.10 m; weight: 77.91 ±15.01 kg; body mass index (BMI): 24.50 ±2.52 kg/m^2^) participated in the data collection performed at the Virtual Reality Laboratory of the VUmc Amsterdam. Subjects provided written informed consent prior to their voluntary participation in the study and after receiving detailed information about the study. Ethical approval was provided by the Scientific and Ethical Review Board (Dutch: Vaste Commissie Wetenschap en Ethiek - VCWE) of the Faculty of Behavior & Movement Sciences, VU University Amsterdam.

### Instrumentation

**Fig. 1:**
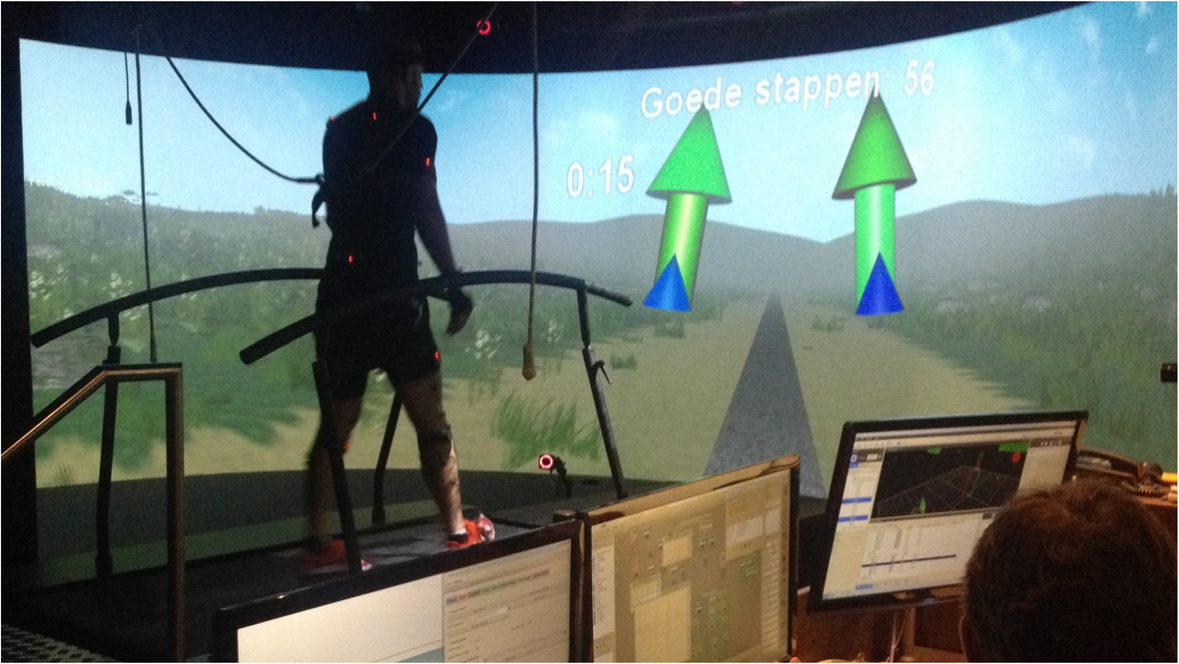
Virtual reality laboratory equipped with a GRAIL system. The subject receives a target foot progression angle (FPA) through an arrow, which changes color, from red to green, depending on the performed angle

Human movement analysis was performed in a Gait Real-time Analysis Interactive Lab (GRAIL, MOTEK BV, Amsterdam, NL) depicted in Fig. 1. The GRAIL system is composed of a dual-belt instrumented treadmill with two full 6D force plates beneath each belt capturing at 1000 Hz. In addition, the system features a ten-camera system tracking 22 passive reflective markers at 100 Hz (Vicon, Oxford Metrics Group, Oxford, UK). Markers were placed on the following body locations according to the lower body configuration of Human Body Model 2: anterior and posterior superior iliac spine, medial and lateral femoral epicondyle, medial and lateral malleolus, second metatarsal, fifth metatarsal, calcaneus, lateral mid-shank, and lateral mid-thigh. To enable the laboratory feedback, a semi-cylindrical screen located anterior to the treadmill was utilized that projected an immersive virtual reality environment. Integration and control of the GRAIL components is enabled by the D-Flow software package [45], and real-time biomechanical modeling was performed through the Human Body Model (HBM) software package [31, 46].

Concurrently with the GRAIL measurements, Xsens MVN Awinda inertial motion capture system (Xsens Technologies BV, Enschede, NL) [47] was used with the lower body configuration. Seven Xsens MTw IMUs with dimensions 47 x 30 x 13 mm and orientation dynamic accuracy 0.75 deg RMS for roll/pitch and 1.5 deg for heading components were used. The full scales of the measurement units are ±160*m*/*s*^2^ for the accelerometer, ±2000*deg*/*s* for the gyroscope, and ±1.9*Gauss* for the magnetometer [48]. Five IMUs were mounted on pelvis, thighs, and shanks using the accompanying Velcro straps and two IMUs were placed on feet by firmly tying them with the laces on each participant’s own shoes. The software version of Xsens MVN Analyze 2018.0 was used to reconstruct the lower body kinematics at 60 Hz [26]. The software features consistent behavior, even at the presence of magnetic disturbances, making it suitable for use on a treadmill and any other environment regardless of its magnetic field homogeneity. Segment orientations were obtained through the software by applying the IMU-to-segment alignment, found using an a-priori-known upright pose (N-pose) performed by the subject during the calibration [49]. The second part of the calibration of Xsens MVN consisted of comfortable walking in a straight line for approximately 5 meters. The output of the Xsens MVN Analyze is three-dimensional positions and orientations of the modeled body segments, expressed in an external coordinate frame defined during the calibration [26].

**Fig. 2:**
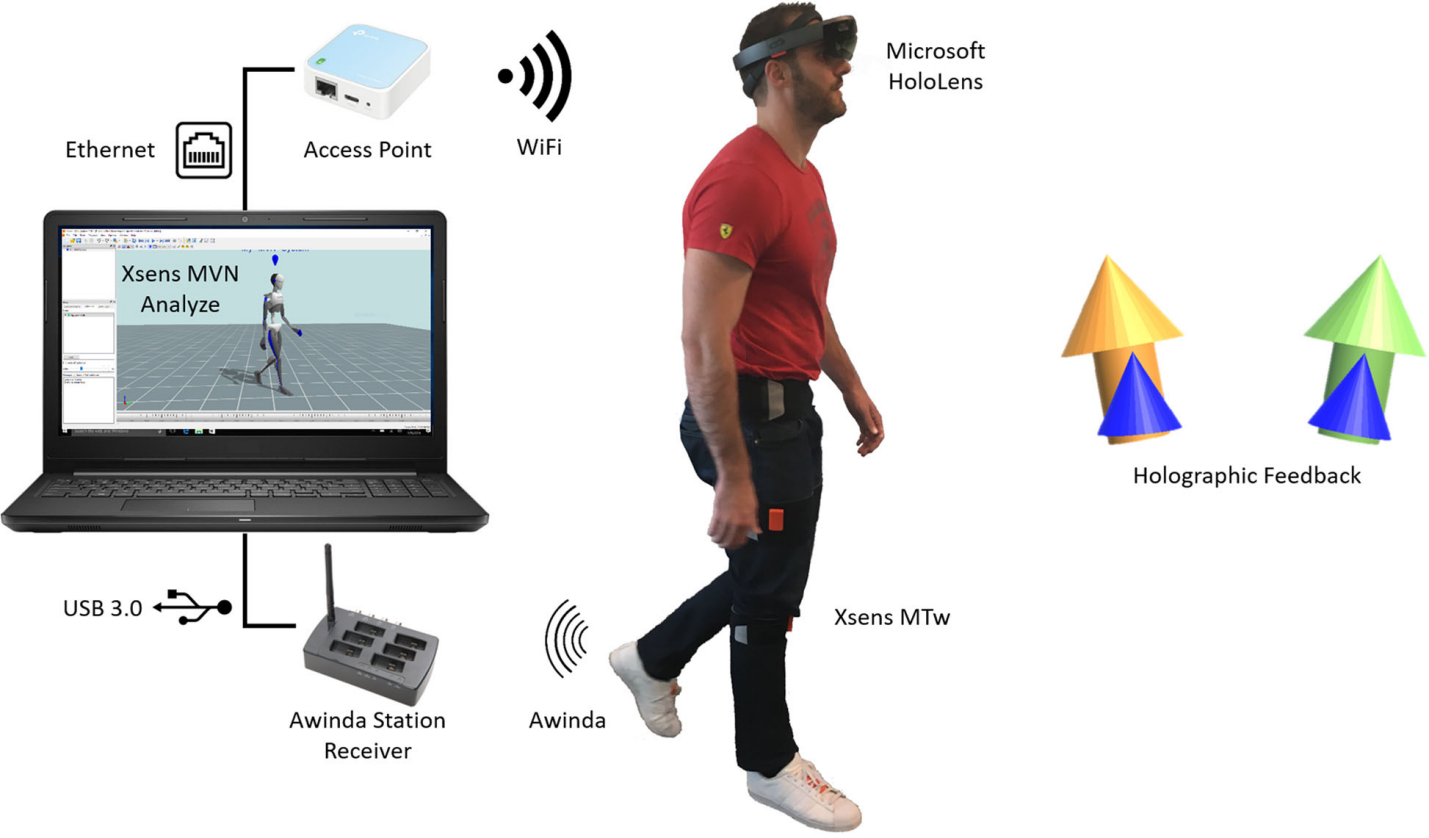
Wearable biofeedback setup. Xsens MVN Analyze receives the MTw sensor data via the Xsens Awinda Station, reconstructs the lower body kinematics, and streams via UDP. Microsoft HoloLens receives the kinematic input via Wi-Fi, calculates the foot progression angle, and updates the holographic feedback visualization

To enable the wearable biofeedback, Microsoft HoloLens was used (Microsoft Corp., Redmond, WA, USA) [44]. This wearable augmented reality headset device is capable of projecting holograms (three-dimensional visualizations) in the environment of use. The biofeedback was developed as a Universal Windows Platform (UWP) application built in Unity 3D Game Engine version 5.6.2 (Unity Technologies SF, San Francisco, CA, USA), and receives kinematic input (packet size = 760 bytes) from Xsens MVN Analyze, in real-time, via User Datagram Protocol (UDP) at 30 Hz. Networking of the devices was configured via an access point (TP-Link TL-WR802N, 300MBit/s, 2.4GHz), which was connected to the computer running Xsens MVN Analyze via Ethernet and to the Microsoft HoloLens via Wi-Fi. The components comprising the wearable biofeedback system are illustrated in Fig. 2.

The biofeedback was visualized similarly on both laboratory and wearable screen, in accordance with a previous gait retraining study involving patients of KOA [12]. Figure 2 illustrates the wearable biofeedback setup, in which the feedback object is a blue cone with 2D orientation updated based on the FPA per step. The target object is an arrow placed behind the feedback cone, the color of which is updated depending on the agreement between the estimated and target FPA. More specifically, the color changes were based on the absolute difference between target and performed FPA: green when |FPA-target |≤2°, yellow when 2°<|FPA-target |≤5°, and red when |FPA-target |>5°. These are arbitrary chosen values, with the green range matching targets used in previous studies [12, 36, 41].

In the Microsoft HoloLens visualizations, billboarding and tag along features were added to the holograms to update their position, such that they would always face the user and only translate when they were entirely outside the user’s field of view. These techniques ensure availability of the content at all times while minimizing the unpleasant effects of visualizations that are tighly coupled to the motion of the headset (head-locked content) [50].

For practical reasons, the Microsoft HoloLens application featured speech command capabilities to enable the initialization of the training protocol. Thus, right after the initialization of the treadmill belt, the researcher performing the experiment approached the subject who was already walking on the treadmill to provide the triggering key-phrase “go to mode zero” close to the microphone of the Microsoft HoloLens.

### Experimental procedures

Subsequently, placement of the reflective markers and IMUs was performed, followed by calibration of Xsens MVN system. As a last preparation step, participants performed a T-pose and walked a few steps on the treadmill to anatomically calibrate the optical motion capture system.

The first series of experiments comprised treadmill walking at a preselected constant speed of 1.2 m/s for 13.5 minutes. An acclimatization period with no target was provided for one minute. Next, the following five target angles were projected in a random order, for two minutes each: 15, 10, 5 degrees toe-out, 0 degrees straight toes, and 5 degrees toe-in. Visual feedback on the performed FPA was provided on each performed step on the laboratory screen. Before any new target, a 30-second active rest period was provided, during which subjects kept walking without receiving any target.

**Fig. 3:**
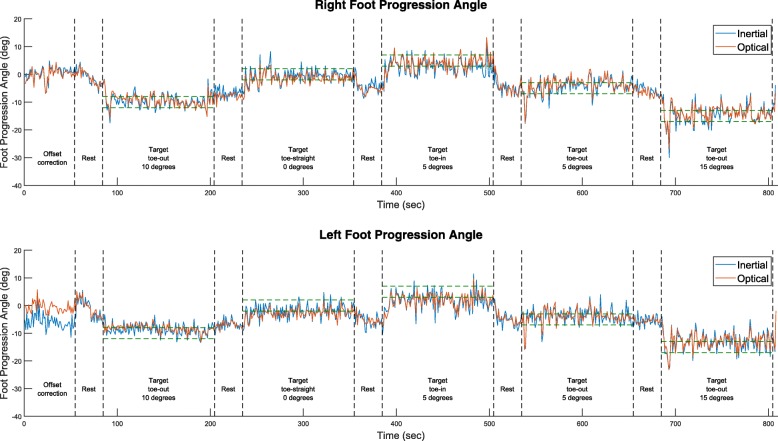
Illustration of the results for right and left foot progression angles across the whole training protocol, estimated at each entire foot contact via inertial (blue lines) or optical (red lines) motion capture input. An offset correction is calculated during the first 60 s when participants are instructed to walk with straight toes and applied after that. A unique random target is provided for 120 s (mid-point of green dashed lines indicating the ±2° good step range), after 30 s of no target (rest) period

During the second series of experiments, participants wore the Microsoft HoloLens for a brief period to familiarize with the device. The complete protocol was repeated for another 13.5 minutes, by projecting the targets and IMU-driven feedback only on the wearable screen of the Microsoft HoloLens. An additional calibration step was introduced, where participants were asked to walk with their toes pointing straight for one minute to detect and reduce any heading offsets introduced by the inertial motion capture system. The average FPA estimated by the wearable system during this period was subtracted from the FPA values estimated in the rest of the trial. An example of the feedback protocol with the targeted and performed FPA values, and the various modes across the full duration of the protocol is illustrated in Fig. 3.

### Computational procedures

The inertial motion capture system outputs global positions and orientations of the tracked body segments. The toe and heel positions are used to calculate the FPA of the *i*th step, when the foot is placed approximately horizontally on the treadmill belt. Assuming heel strike at initial foot contact, entire contact of a foot is identified at the timepoint when the magnitude of heel and toe velocities is close to zero, empirically found as the magnitude of the first derivative of toe position (***p***_*t*_) and heel position (***p***_*h*_), $\left |\boldsymbol {\dot {p}_{t}}\right | < 0.2$ m/s and $\left |\boldsymbol {\dot {p}_{h}}\right | < 0.2$ m/s, respectively.

We define the foot vector for the *ith* step (***r***_*f*,*i*_) as the line from heel (***p***_*h*,*i*_) to toe (***p***_*t*,*i*_) during phase of entire foot contact:

1 $$  \boldsymbol{r}_{f,i} = \boldsymbol{p}_{t,i} - \boldsymbol{p}_{h,i}  $$

Similarly, the heading vector of the *ith* step (***r***_*w*,*i*_) is defined as the displacement vector between the position of the heel in two successive steps:

2 $$ \boldsymbol{r}_{w,i} = \boldsymbol{p}_{h,i} - \boldsymbol{p}_{h,i-1}  $$

**Fig. 4:**
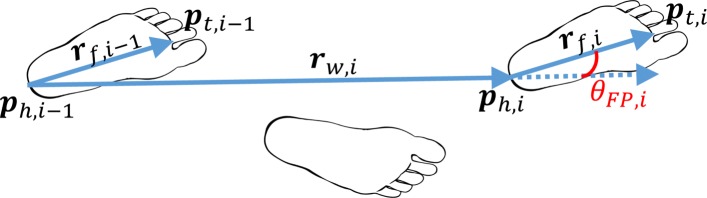
Transverse caudal view of the feet, illustrating the calculation of the foot progression angle for the right foot. The foot progression angle (*θ*_*FP*_) of the *i*th step is derived from the difference of foot vector (*r*_*f*_) and heading vector (*r*_*w*_). The latter two vectors are computed based on the positions of the heel (*p*_*h*_) and toe (*p*_*t*_) as illustrated in the figure

FPA is calculated as the difference between the foot and the heading vectors projected on the transverse plane (Fig. 4), defined by anterior (*x*) and lateral (*y*) axes:

3 $$ {\theta}_{FP,i} = arctan2\left(\frac{r_{w,i,x}}{r_{w,i,y}}\right)-arctan2\left(\frac{r_{f,i,x}}{r_{f,i,y}}\right)  $$

In the laboratory system, FPA is calculated and averaged within a time interval, when the vertical ground reaction force recorded by the respective plate, is greater than a threshold of 10N. Toe and heel positions correspond to the markers placed on the second metatarsal and calcaneus, respectively and the foot vector is calculated based on Eq. 1. Differently to the wearable system, the heading vector is constant and defined as the anterior axes of the lab coordinate system aligned with the belt of the treadmill.

### Data Processing and Statistical Analysis

Data analysis focused on evaluating the performance of the wearable system in estimating FPA, in real-time versus the laboratory system, and to quantify the effectiveness of the two feedback modalities. Firstly, we compared the calculated FPA using the inertial motion capture system versus the optical motion capture system during the second series of experiments with the wearable feedback. The root-mean-squared differences were computed per target angle. Pearson’s *r*^2^ correlation and two-way random single measures intraclass correlation (ICC) were used to to quantify the agreement and consistency between the two estimation systems. Secondly, we examined the effectiveness of the wearable biofeedback system versus the established laboratory solution in altering the user’s FPA. To quantify the effectiveness, we analyzed the number and percentage of good steps, defined as the steps with FPA within the ±2 degrees tolerance range as suggested in the literature [38, 41]. An analysis of variance (ANOVA) across all target conditions and systems, with Tukey’s post-hoc analysis was performed to test whether the performed FPAs differ significantly across target conditions. Significance level was set to 0.05 and confidence interval at 95%. Data analysis was performed in MATLAB 2017a.

## Results

**Fig. 5:**
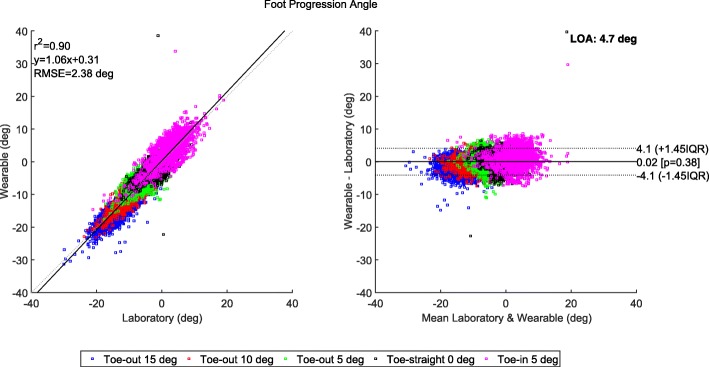
Correlation and Bland-Altman plots for the foot progression angle estimates based on the wearable and laboratory setup

Correlation and Bland-Altman plots are shown in Fig. 5. Correlation coefficients were found of 0.9 for *r*^2^ and of 0.94 for ICC. Accuracy analysis across all data points showed RMS difference of 2.38 degrees and level of agreement (LOA) about 4.7° between the wearable and laboratory estimates. Per target mode, RMS difference (average across subjects ± standard deviation) was found to be 2.25±1.10, 2.18±0.90, 2.02±0.90, 2.62±1.22, 1.86±0.73 degrees for target FPAs of −15, −10, −5, −0, and 5 degrees, respectively.

During the first minute of the wearable feedback session, when subjects were instructed to walk with straight toes, mean FPA values equaled −1.61±2.47° as recorded by the laboratory system, and −1.96±2.91° as recorded by the wearable system.

**Fig. 6:**
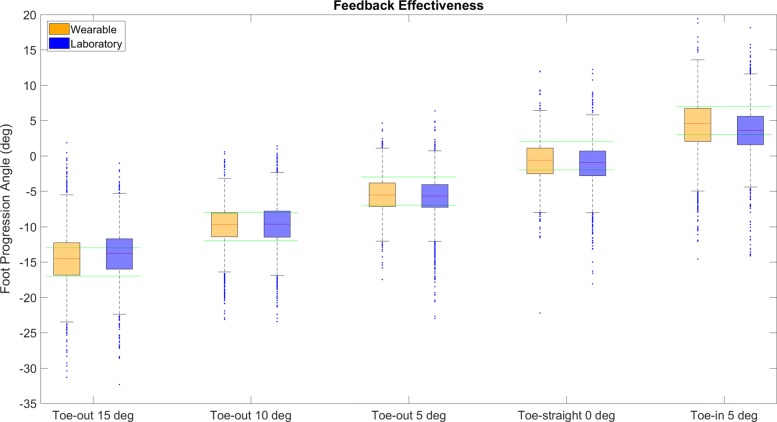
Box plot of all steps per target mode in the wearable and laboratory feedback. Green lines are the target limits of each mode

Figure 6 shows the box plots per target mode and per feedback scheme. The mean ± standard deviations of the differences from the targets for the wearable feedback were 0.48 ±3.75°, 0.07 ±2.86°, −0.46±2.57°, −0.78±3.01°, −0.83±3.97° and for the laboratory feedback 1.04 ±3.44°, 0.28±3.13°, −0.91±3.03°, −1.20±3.07°, −1.49±3.50° for −15°, −10°, −5°, 0°, and 5° target FPA, respectively. In both systems multivariate ANOVA test showed significant differences between the FPAs of each target mode regardless of system used for the feedback, while post-hoc analysis across the five different modes showed that the FPAs during each target mode differed significantly to other target modes (*p*< 0.001).

**Fig. 7:**
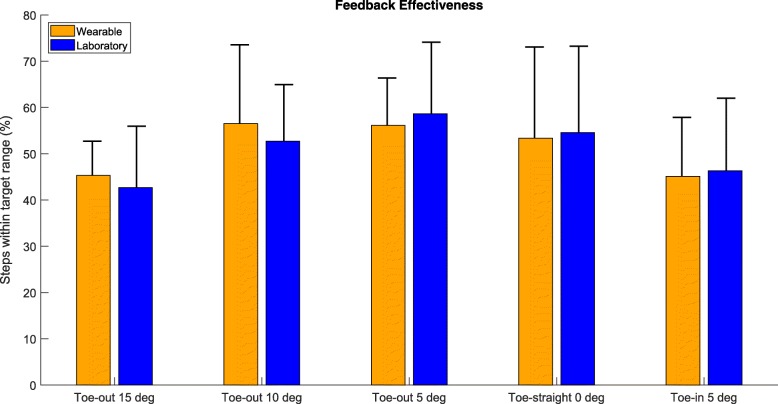
Bar plot illustrating the mean and standard deviation of the percentage of good steps (± 2 degrees from the target), across 11 subjects, for wearable (orange) and laboratory (blue) feedback setups

Feedback effectiveness based on the percentage of good steps with FPA within the ± 2° range is illustrated in Fig. 7. Percentage of good steps in the laboratory feedback was 51±15*%* across 12033 steps over all subjects and targets. Per target percentage of good steps was found to be 42.7 ± 13.2*%*, 52.7 ± 12.2*%*, 58.6± 15.5*%*, 54.5± 18.7*%*, 46.3± 15.7*%* for FPAs of −15, −10, −5, 0, and 5 degrees, respectively. In the case of FPA feedback provided and calculated in the wearable setup, an overall percentage of good steps 48.3 ± 12.8*%* across 12075 steps was found. For the aforementioned ascending order of targets, effectiveness per mode was observed to be of 39.4 ± 9.2*%*, 54.4 ± 14.9*%*, 54.3 ± 11.0*%*, 51.4 ± 15.1*%*, 42.1 ± 13.9*%*. When the FPAs of the wearable feedback were calculated from the optical motion capture system, the percentage of good steps was found overall 51.3± 13.4*%*, with individual per mode effectiveness of 45.3 ± 7.4*%*, 56.5 ± 17.0*%*, 56.1 ± 10.3*%*, 53.3 ± 19.7*%*, 45.1 ± 12.7*%*.

## Discussion

In this study we proposed a method to perform gait retraining of the FPA using real-time biofeedback based entirely on wearable sensing and feedback modules. To our knowledge, this is the first study investigating a fully wearable visual feedback system for the purpose of retraining the FPA. Our findings demonstrated that FPA estimates derived from the inertial motion tracking input matched closely the ones from optical motion capture, with an overall RMS difference of 2.38 degrees. In addition, when incorporating a wearable augmented reality headset, the biofeedback effectiveness, based on steps within a ± 2° target range, matched closely the laboratory approach.

Our accuracy analysis depends on an optical motion capture system reference. However, previous studies have indicated that orientations of the transverse plane may also suffer from inter-trial, inter-session, and inter-observer differences. In particular, for the foot heading angle, median within-assessor reliability across four studies [51–54] was reported to provide multiple correlation coefficient of 0.55 [55]. In addition, the same systematic review discussed five other studies that reported inter-assessor standard deviation of the foot progression that ranged from 2 to 5 degrees [56–60]. These literature findings suggest that error magnitudes of around 2 degrees, as found in our study, are typically found in conventional optical motion capture systems, as a result of marker placement, computational method, measurement system accuracy and resolution, or observer’s experience and skills. Therefore, given that these measurement errors are considered clinically acceptable, our method is sufficiently accurate in tracking FPA for the specific application.

In our study we used a sensor set of seven IMUs required by Xsens MVN software to track both positions and orientations of the feet and other lower body segments. A question arises whether fewer IMUs would suffice for this task. Related studies have proposed a set of one sensor per foot combined with a magneto-inertial sensor fusion algorithm to derive the FPA [27, 61]. Even though those studies noted no effect of magnetic disturbances in the estimates, it has been previously shown that inertial-magnetic motion tracking is affected by the homogeneity of the magnetic field [62]. In addition, the use on a treadmill, which typically contain several electromagnetic components beneath the belt, would create a non-homogeneous magnetic field. As a result, an approach heavily relying on magnetometers would suffer from orientation drift over time. The present study used the latest version of Xsens MVN software, which provides a consistent pose of the body pose regardless of magnetic disturbances in the environment of use [26].

Calculation of the foot vector angle using both inertial and optical motion capture solutions may suffer from offsets of approximately 1–3 degrees, due to measurement and modeling error in both approaches. In inertial motion capture, offsets in the foot vector may be introduced as a result of a mismatch between the modeled and practiced N-pose used to calibrate the system. Similarly, optical motion capture may be susceptible to sub-centimeter misplacement of the markers on the second metatarsal and calcaneus. For instance, misplacements of markers on the foot may result in erratic estimation of the foot vector. In the accuracy analysis we subtracted these offsets, based on the median FPA during the first one minute of the trial. Moreover, gait event detection methods differ between both systems and may introduce differences. The wearable system relies on detection of near-zero velocity to detect contact with the ground which may be sensitive to the walking speed and style. In contrast, the laboratory system is based on force plate detection, which may be erratic in real-time in case the subject steps on the contra-lateral force plate. In the offline analysis we have corrected for these cases, by calculating the gait events based on marker velocity. Another source of difference in the laboratory system is that for convenience, the heading vector was set constant, aligned with the anterior axis of the global coordinate system matching the movement direction of the belt. In other words, contrary to the wearable system, differences in walking direction are not taken into account in the FPA calculation of the laboratory system.

A major advantage of the proposed wearable system compared to laboratory-based setups is its significantly lower cost. Fully functional virtual reality laboratories typically cost between tens to hundreds thousand dollars, depending on the type of optical motion capture systems, instrumented treadmills, and immersive environment systems. The setup proposed in this study is composed of an augmented reality headset and inertial sensors. Current price of the development version of Microsoft HoloLens is around 3000 dollars, while the cost of goods for inertial sensor components (accelerometer, gyroscope, and magnetometer) has nowadays dropped to a few tens of dollars per module. Additional costs may include the cost for networking devices, computers and software. Costs for software vary considerably and are therefore difficult to quantify, since they usually depend on development efforts, number of users and other market-driven factors [63].

Besides costs, the proposed method based on a set of IMUs and an AR headset reduces the complexity and increases the flexibility of gait retraining methods significantly compared to conventional laboratory techniques. Alternative approaches with lower cost and complexity have been previously proposed, even without the necessity for electronic equipment. For instance, mirror-based biofeedback for FPA retraining of patients with knee osteoarthritis has been investigated by Hunt et al. [33]. That study reported significantly lower performance of the mirror feedback compared to real-time visual biofeedback, with mean differences of approximately 2 degrees. However, despite the significantly lower performance of the mirror and given the high costs of laboratory-based biofeedback setups, the former was favored as an acceptable solution for clinical practice. Our work provides a method that matches closely the performance of laboratory feedback systems in terms of both tracking accuracy and feedback effectiveness, while reducing the costs and increasing the portability and potential of performing gait retraining in any environment. In addition, recent studies reported that subject-specific gait modifications decrease knee joint loading significantly more, compared to generalized targets [64]. Therefore, performance and portability of motion tracking and biofeedback are both important factors in retaining the decrease of joint loading, effectively in each individual patient.

The feedback effectiveness of both wearable and laboratory systems is affected by the arbitrary chosen value of ± 2°. This has been a point of discussion also by Chen et al. [41] when applying a vibrotactile feedback setup driven by marker-based motion capture. Further research is required to identify optimal thresholds for the green, yellow and red zones of the visualization. Moreover, in this study we have used visualization of the target and feedback values as arrows and cones able to rotate. Alternative visualizations such as in [31] should be examined to find the ideal visualization method for gait retraining of the FPA with visual biofeedback.

Inertial sensing and augmented reality technologies have both advanced vastly in the last years. Their unique combination enables not only applications of rehabilitation, such as gait retraining, but could potentially be expanded to other fields, such as live entertainment and gaming using input from a user’s own body motion to drive graphics in a mixed reality environment. Devices such as the Microsoft HoloLens utilize a number of sensors to derive its own position and orientation in space. All heavy computations needed for self-localization and visualization of the holograms in space are executed standalone in real-time. In addition, these kinematic estimates can be fused with inertial motion capture to correct any position drift introduced by the latter, for instance due to errors in the measured segment lengths. The downsides of the first commercially available and latest to date version of the headset are that it is rather heavy (approx. 0.5 kg), bulky, and impractical for use in daily life. Moreover, the field of view is currently narrow. Upcoming developments in augmented reality headsets are expected to improve the functionality for potentially unobtrusive daily life use in the future.

We evaluated the method on a treadmill, however, the wearable setup enables the application of gait retraining in overground walking that differs in terms of gait mechanics and metabolic energy cost [65]. As discussed previously the use of more sensors may reduce the drift over time, however, the system may not be comfortable for uses of long duration during daily living. Further research towards magnetically immune motion tracking systems that require fewer sensors is necessary, to achieve reliable orientation estimates regardless the environmental conditions or movement performed. Leveraging the increased practicality of fewer sensors and consistent performance over time could enable daily life applications, requiring continuous monitoring of important kinematic parameters.

In our study, awareness of the distance from the FPA target was considered an important advantage of visual feedback with respect to alternative modalities, such as tactile and auditory. Wheeler et al. compared both visual and tactile feedback, reporting that despite both being equally effective, visual feedback required less time from subjects to converge to the targeted gait pattern. However, whether quantitative information can actually boost the feedback effectiveness remains unknown. Another advantage of visual feedback compared to other modes are the gamification prospects, which could motivate subjects to perform the training in a game-like fashion. Nevertheless, further comparison studies between various feedback modalities should be performed to assess the most effective and most engaging type of biofeedback.

In our study we examined the wearable sensing and feedback in a group of young healthy adults, similarly to previous studies evaluating experimental technology for gait retraining [7, 28, 31, 32, 35, 37–39, 41, 42, 66]. However, the eventual application is targeted to patients of KOA who are generally older and less familiar with technology. Further studies to evaluate the applicability of the system in patients with KOA is required. Potential issues that may be met with the current setup is the inability of patients to perform the N-pose due to increased static knee varus/valgus type malalignment with an unknown effect to the FPA estimate. Manual input of the joint angles performed during the static calibration trial may be an appropriate solution for this. Nevertheless, the portability of the system could facilitate applications initially in clinical environments with the help of a medical specialist, and subsequently for home use. In particular, such setup allows for increased number of training sessions, which may result in enhanced training retention over time. Moreover, combining such home retraining system with telemedicine techniques could enable objective data for remote monitoring of the gait pattern of patients and identifying changes over time.

## Conclusion

This study investigated the feasibility, accuracy, and effectiveness of combining a commercially available inertial motion capture system and an augmented reality headset to perform gait retraining to alter the FPA. The findings proved sufficient accuracy of the FPA estimates with the ones obtained from optical motion capture. At the same time, average feedback effectiveness based on number of steps within a ± 2° range from the target was found around 50% for both setups. The proposed setup is completely wearable and enables gait retraining applications in clinical settings without the need for a complex gait motion analysis laboratory. For daily monitoring of FPA, further developments towards reduced sensor setups with immunity to magnetic disturbances are recommended.

## Abbreviations

AR: Augmented reality
BMI: Body mass index
FPA: Foot progression angle
GRAIL: Gait real-time analysis interactive lab
HBM: Human body model
IMU: Inertial measurement unit
KOA: Knee osteoarthritis
RMS: Root mean square
UDP: User datagram protocol
UWP: Universal windows platform

## References

[1] Woolf AD, Pfleger B (2003). Burden of major musculoskeletal conditions. Bull World Health Organ.

[2] Felson DT, Lawrence RC, Hochberg MC (2000). Osteoarthritis: New insights. part 2: treatment approaches. Ann Intern Med.

[3] Conaghan PG, Dickson J, Grant RL (2008). Care and management of osteoarthritis in adults: summary of nice guidance. BMJ.

[4] Boutron I, Tubach F, Giraudeau B, Ravaud P (2003). Methodological differences in clinical trials evaluating nonpharmacological and pharmacological treatments of hip and knee osteoarthritis. JAMA.

[5] Sarzi-Puttini P, Cimmino MA, Scarpa R, Caporali R, Parazzini F, Zaninelli A, Atzeni F, Canesi B (2005). Osteoarthritis: An overview of the disease and its treatment strategies. Semin Arthritis Rheum.

[6] Reeves ND, Bowling FL (2011). Conservative biomechanical strategies for knee osteoarthritis. Nat Rev Rheumatol.

[7] Barrios JA, Crossley KM, Davis IS (2010). Gait retraining to reduce the knee adduction moment through real-time visual feedback of dynamic knee alignment. J Biomech.

[8] Fregly BJ, Reinbolt JA, Rooney KL, Mitchell KH, Chmielewski TL (2007). Design of patient-specific gait modifications for knee osteoarthritis rehabilitation. IEEE Trans Biomed Eng.

[9] Shull PB, Jirattigalachote W, Hunt Ma, Cutkosky MR, Delp SL (2014). Quantified self and human movement: A review on the clinical impact of wearable sensing and feedback for gait analysis and intervention. Gait Posture.

[10] Jackson B, Wluka A, Teichtahl A, Morris M, Cicuttini F (2004). Reviewing knee osteoarthritis—a biomechanical perspective. Sci Med Sport.

[11] Zhao D, Banks SA, Mitchell KH, D&apos;Lima DD, Colwell CW, Fregly BJ (2007). Correlation between the knee adduction torque and medical contact force for a variety of gait patterns. J Orthop Res.

[12] Richards RE, van den Noort JC, van der Esch M, Booij MJ, Harlaar J (2018). Effect of real-time biofeedback on peak knee adduction moment in patients with medial knee osteoarthritis: Is direct feedback effective?. Clin Biomech.

[13] Lin C-J, Lai K-A, Chou Y-L, Ho C-S (2001). The effect of changing the foot progression angle on the knee adduction moment in normal teenagers. Gait Posture.

[14] Mündermann A, Dyrby CO, Hurwitz DE, Sharma L, Andriacchi TP (2004). Potential strategies to reduce medial compartment loading in patients with knee osteoarthritis of varying severity: reduced walking speed. Arthritis Rheumatol.

[15] Chang A, Hurwitz D, Dunlop D, Song J, Cahue S, Hayes K, Sharma L (2007). The relationship between toe-out angle during gait and progression of medial tibiofemoral osteoarthritis. Ann Rheum Dis.

[16] Guo M, Axe MJ, Manal K (2007). The influence of foot progression angle on the knee adduction moment during walking and stair climbing in pain free individuals with knee osteoarthritis. Gait Posture.

[17] Rutherford D, Hubley-Kozey C, Deluzio K, Stanish W, Dunbar M (2008). Foot progression angle and the knee adduction moment: a cross-sectional investigation in knee osteoarthritis. Osteoarthr Cartil.

[18] Simic M, Wrigley TV, Hinman RS, Hunt Ma, Bennell KL (2013). Altering foot progression angle in people with medial knee osteoarthritis: The effects of varying toe-in and toe-out angles aremediated by pain and malalignment. Osteoarthr Cartil.

[19] Shull PB, Shultz R, Silder A, Dragoo JL, Besier TF, Cutkosky MR, Delp SL (2013). Toe-in gait reduces the first peak knee adduction moment in patients with medial compartment knee osteoarthritis. J Biomech.

[20] Hunt MA, Takacs J (2014). Effects of a 10-week toe-out gait modification intervention in people with medial knee osteoarthritis: A pilot, feasibility study. Osteoarthr Cartil.

[21] Bergmann J, McGregor A (2011). Body-worn sensor design: what do patients and clinicians want?. Ann Biomed Eng.

[22] Kainz H, Graham D, Edwards J, Walsh HP, Maine S, Boyd RN, Lloyd DG, Modenese L, Carty CP (2017). Reliability of four models for clinical gait analysis. Gait Posture.

[23] Andersen MS, Damsgaard M, Rasmussen J (2009). Kinematic analysis of over-determinate biomechanical systems. Comput Methods Biomech Biomed Eng.

[24] Luinge HJ, Veltink PH (2005). Measuring orientation of human body segments using miniature gyroscopes and accelerometers. Med Biol Eng Comput.

[25] Roetenberg D, Luinge H, Slycke P. Xsens MVN: full 6DOF human motion tracking using miniature inertial sensors. Xsens Motion Technol BV Tech Rep. 2009;:1–9.

[26] Schepers HM, Giuberti M, Bellusci G. Xsens MVN: Consistent Tracking of Human Motion Using Inertial Sensing. Tech Rep Xsens Technol B V. 2018;:1–8.

[27] Huang Y, Jirattigalachote W, Cutkosky MR, Zhu X, Shull PB (2016). Novel Foot Progression Angle Algorithm Estimation via Foot-Worn, Magneto-Inertial Sensing. IEEE Trans Biomed Eng.

[28] Xu J, Bao T, Lee UH, Kinnaird C, Carender W, Huang Y, Sienko KH, Shull PB (2017). Configurable, wearable sensing and vibrotactile feedback system for real-time postural balance and gait training: proof-of-concept. J Neuroengineering Rehabil.

[29] Richards R, van den Noort JC, Dekker J, Harlaar J (2017). Gait retraining with real-time biofeedback to reduce knee adduction moment: Systematic review of effects and methods used. Arch Phys Med Rehabil.

[30] Hunt MA, Bennell KL (2011). Predicting dynamic knee joint load with clinical measures in people with medial knee osteoarthritis. Knee.

[31] van den Noort JC, Steenbrink F, Roeles S, Harlaar J. Real-time visual feedback for gait retraining: toward application in knee osteoarthritis. Med Biol Eng Comput. 2014;:275–86. 10.1007/s11517-014-1233-z. 10.1007/s11517-014-1233-z 25480419

[32] Hunt MA, Simic M, Hinman RS, Bennell KL, Wrigley TV (2011). Feasibility of a gait retraining strategy for reducing knee joint loading: Increased trunk lean guided by real-time biofeedback. J Biomech.

[33] Hunt MA, Takacs J, Hart K, Massong E, Fuchko K, Biegler J (2014). Comparison of Mirror, Raw Video, and Real-Time Visual Biofeedback for Training Toe-Out Gait in Individuals With Knee Osteoarthritis. Arch Phys Med Rehabil.

[34] Segal NA, Glass NA, Teran-Yengle P, Singh B, Wallace RB, Yack HJ. Intensive Gait Training for Older Adults with Symptomatic Knee Osteoarthritis. Am J Phys Med Rehabil. 2015;1. 10.1097/PHM.0000000000000264. 10.1097/PHM.0000000000000264 PMC4567520 25768068

[35] Pizzolato C, Reggiani M, Saxby DJ, Ceseracciu E, Modenese L, Lloyd DG (2017). Biofeedback for gait retraining based on real-time estimation of tibiofemoral joint contact forces. IEEE Trans Neural Syst Rehabil Eng.

[36] Richards R, van der Esch M, van den Noort JC, Harlaar J (2018). The learning process of gait retraining using real-time feedback in patients with medial knee osteoarthritis. Gait Posture.

[37] Wheeler JW, Shull PB, Besier TF (2011). Real-Time Knee Adduction Moment Feedback for Gait Retraining Through Visual and Tactile Displays. J Biomech Eng.

[38] Shull PB, Lurie KL, Cutkosky MR, Besier TF (2011). Training multi-parameter gaits to reduce the knee adduction moment with data-driven models and haptic feedback. J Biomech.

[39] Dowling AV, Fisher DS, Andriacchi TP (2010). Gait Modification via Verbal Instruction and an Active Feedback System to Reduce Peak Knee Adduction Moment. J Biomech Eng.

[40] Shull PB, Silder A, Shultz R, Dragoo JL, Besier TF, Delp SL, Cutkosky MR (2013). Six-week gait retraining program reduces knee adduction moment, reduces pain, and improves function for individuals with medial compartment knee osteoarthritis. J Orthop Res.

[41] Chen DKY, Haller M, Besier TF. Wearable lower limb haptic feedback device for retraining foot progression angle and step width. Gait Posture. 2017;55; Supplement C:177–83. 10.1016/j.gaitpost.2017.04.028. 10.1016/j.gaitpost.2017.04.028 28460321

[42] Ferrigno C, Stoller IS, Shakoor N, Thorp LE, Wimmer MA (2016). The feasibility of using augmented auditory feedback from a pressure detecting insole to reduce the knee adduction moment: a proof of concept study. J Biomech Eng.

[43] Seuter M, Opitz L, Bauer G, Hochmann D. Live-feedback from the imus: Animated 3d visualization for everyday-exercising. In: Proceedings of the 2016 ACM International Joint Conference on Pervasive and Ubiquitous Computing: Adjunct. ACM: 2016. p. 904–7.

[44] The leader in Mixed Reality Technology – HoloLens. https://www.microsoft.com/en-us/hololens. Accessed 25 Aug 2017.

[45] Geijtenbeek T, Steenbrink F, Otten B, Even-Zohar O. D-flow: immersive virtual reality and real-time feedback for rehabilitation. In: Proceedings of the 10th International Conference on Virtual Reality Continuum and Its Applications in Industry. ACM: 2011. p. 201–8.

[46] Van den Bogert AJ, Geijtenbeek T, Even-Zohar O, Steenbrink F, Hardin EC (2013). A real-time system for biomechanical analysis of human movement and muscle function. Med Biol Eng Comput.

[47] Xsens MVN Analyze - Products - Xsens 3D motion tracking. https://www.xsens.com/products/xsens-mvn-analyze/. Accessed 23 Dec 2017.

[48] Xsens MTw Awinda - Products - Xsens 3D motion tracking. https://www.xsens.com/products/mtw-awinda/. Accessed 07 Apr 2018.

[49] Xsens MVN User Manual. https://xsens.com/download/usermanual/3DBM/MVN_User_Manual.pdf. Accessed 01 May 2017.

[50] Billboarding and tag-along - Windows Mixed Reality - Microsoft. https://developer.microsoft.com/en-us/windows/mixed-reality/billboarding_and_tag-along. Accessed 24 Jan 2018.

[51] Growney E, Meglan D, Johnson M, Cahalan T, An K-N (1997). Repeated measures of adult normal walking using a video tracking system. Gait Posture.

[52] Kadaba M, Ramakrishnan H, Wootten M, Gainey J, Gorton G, Cochran G (1989). Repeatability of kinematic, kinetic, and electromyographic data in normal adult gait. J Orthop Res.

[53] Steinwender G, Saraph V, Scheiber S, Zwick EB, Uitz C, Hackl K (2000). Intrasubject repeatability of gait analysis data in normal and spastic children. Clin Biomech.

[54] Tsushima H, Morris ME, McGinley J (2003). Test-retest reliability and inter-tester reliability of kinematic data from a three-dimensional gait analysis system. J Jpn Phys Ther Assoc.

[55] McGinley JL, Baker R, Wolfe R, Morris ME (2009). The reliability of three-dimensional kinematic gait measurements: a systematic review. Gait Posture.

[56] Eve L, McNee A, Shortland A (2006). Extrinsic and intrinsic variation in kinematic data from the gait of healthy adult subjects. Gait Posture.

[57] Gorton G, Hebert D, Goode B (2002). Assessment of the kinematic variability between twelve shriners motion analysis laboratories part 2: short-term follow up. Gait Posture.

[58] Gorton GE, Hebert DA, Gannotti ME (2009). Assessment of the kinematic variability among 12 motion analysis laboratories. Gait Posture.

[59] Murphy A, McGinley J, Tirosh O. Reliability of kinematic gait measurements in adult hemiplegic stroke. Proc 12th Ann Gait Clin Mov Anal Soc. 2007.

[60] Schwartz MH, Trost JP, Wervey RA (2004). Measurement and management of errors in quantitative gait data. Gait Posture.

[61] Xia H, Xu J, Wang J, Hunt MA, Shull PB (2017). Validation of a smart shoe for estimating foot progression angle during walking gait. J Biomech.

[62] de Vries WHK, Veeger HEJ, Baten CTM, van der Helm FCT (2009). Magnetic distortion in motion labs, implications for validating inertial magnetic sensors. Gait Posture.

[63] Hill P, Group ISBS. Practical Software Project Estimation: A Toolkit for Estimating Software Development Effort & Duration: McGraw-Hill Education; 2010. https://books.google.gr/books?id=mW6phtfWZ-EC.

[64] Uhlrich SD, Silder A, Beaupre GS, Shull PB, Delp SL (2018). Subject-specific toe-in or toe-out gait modifications reduce the larger knee adduction moment peak more than a non-personalized approach. J Biomech.

[65] Berryman N, Gayda M, Nigam A, Juneau M, Bherer L, Bosquet L (2012). Comparison of the metabolic energy cost of overground and treadmill walking in older adults. Eur J Appl Physiol.

[66] Clark RA, Pua Y-H, Bryant AL, Hunt MA (2013). Validity of the Microsoft Kinect for providing lateral trunk lean feedback during gait retraining. Gait Posture.

